# Reliable Detection of Chemical Warfare Agents Using High Kinetic Energy Ion Mobility Spectrometry

**DOI:** 10.1021/jasms.4c00240

**Published:** 2024-07-16

**Authors:** Christoph Schaefer, Maria Allers, Moritz Hitzemann, Alexander Nitschke, Tim Kobelt, Max Mörtel, Stefanie Schröder, Arne Ficks, Stefan Zimmermann

**Affiliations:** † Institute of Electrical Engineering and Measurement Technology, Department of Sensors and Measurement Technology, Leibniz University Hannover, Appelstr. 9A, 30167 Hannover, Germany; ‡ Bundeswehr Research Institute for Protective Technologies and CBRN Protection, Humboldtstrasse 100, 29633 Munster, Germany

## Abstract

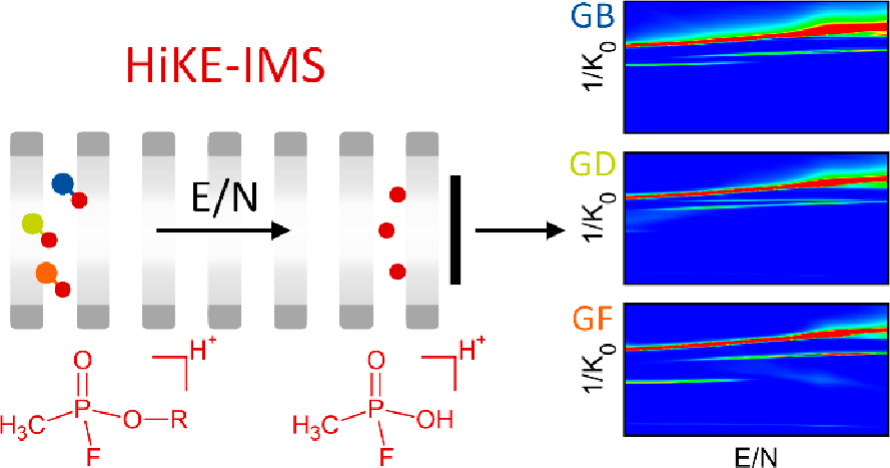

High Kinetic Energy Ion Mobility Spectrometers (HiKE-IMS) ionize and separate ions at reduced pressures of 10–40 mbar and over a wide range of reduced electric field strengths *E*/*N* of up to 120 Td. Their reduced operating pressure is distinct from that of conventional drift tube ion mobility spectrometers that operate at ambient pressure for trace compound detection. High *E*/*N* can lead to a field-induced fragmentation pattern that provides more specific structural information about the analytes. In addition, operation at high *E*/*N* values adds the field dependence of ion mobility as an additional separation dimension to low-field ion mobility, making interfering compounds less likely to cause a false positive alarm. In this work, we study the chemical warfare agents tabun (GA), sarin (GB), soman (GD), cyclosarin (GF) and sulfur mustard (HD) in a HiKE-IMS at variable *E*/*N* in both the reaction and the drift region. The results show that varying *E*/*N* can lead to specific fragmentation patterns at high *E*/*N* values combined with molecular signals at low *E*/*N*. Compared to the operation at a single *E*/*N* value in the drift region, the variation of *E*/*N* in the drift region also provides the analyte-specific field dependence of ion mobility as additional information. The accumulated data establish a unique fingerprint for each analyte that allows for reliable detection of chemical warfare agents even in the presence of interfering compounds with similar low-field ion mobilities, thus reducing false positives.

## Introduction

Chemical warfare agents (CWAs) are characterized by their low lethal dosage combined with the rapid onset of toxic effects. Their chemical properties make them a significant health hazard with the potential to cause significant casualties if they are released to the public.^1^ Although the Chemical Weapons Convention entered into force in 1997, which prohibits their development, production, stockpiling, and use, CWAs have been used in several incidents since then.^1−4^ Accordingly, rapid, sensitive, and reliable methods for the detection of CWAs are still required.

Ion mobility spectrometers (IMS) are widely used in security applications targeting, for example, CWAs,^5−12^ explosives,^13−15^ and drugs of abuse.^16,17^ Most IMS developed for trace compound detection use initially formed reactant ions for the ionization of neutral analyte species in gas-phase ion–molecule reactions in a reaction region. The generated ion population is then separated by their ion mobility in a neutral gas under the influence of an electric field in a drift region. The most common reactant ions in IMS are hydrated hydronium ions H_3_O^+^(H_2_O)_*n*_, ionizing the analytes by proton transfer.^18^ Due to their high proton affinity, many organophosphorus CWAs can be readily ionized by H_3_O^+^(H_2_O)_*n*_.^19^ This results in low limits of detection down to the ppt_V_ (parts per trillion by volume) range in measurement times as short as 1 s.^19^ Although IMS are very sensitive, versatile, and compact instruments for detecting organophosphorus and organosulfur CWAs, they can suffer from a high rate of false alarms due to either spectral interferences or chemical cross-sensitivities during ionization caused by competing ion–molecule reactions between already formed product ions and neutral interfering compounds that lead to a charge loss of the target analyte.^8,20−24^

Classical drift tube ion mobility spectrometers (DTIMS) operate at ambient pressure and separate ions by their ion mobility under “low-field” conditions. The term refers to low electric field strengths that have no significant effect on the energy of the ions. Accordingly, standalone DTIMS are unable to separate ions with the same low-field ion mobility. Therefore, the presence of potentially nonhazardous interfering compound with similar low-field ion mobility as the target analyte can lead to a false positive alarm. For example, triethylamine has a similar ion mobility as sarin and may thus cause a false positive alarm.^8^ In contrast to DTIMS, differential mobility spectrometers (DMS) or field asymmetric ion mobility spectrometers (FAIMS) solely rely on the field dependence of ion mobility and thus cannot separate ions by their low-field ion mobility.^25,26^ Although these techniques can help to distinguish between separate ion species with similar low-field ion mobility, they only determine the differential mobility, i.e., the change in ion mobility between low and high field strengths but not the absolute low and high field ion mobilities.

In contrast to DTIMS and DMS/FAIMS, High Kinetic Energy Ion Mobility Spectrometer (HiKE-IMS) combine the separation of ion species by their low-field ion mobility as in DTIMS with the field dependence of ion mobility as in DMS/FAIMS, while also giving the corresponding absolute ion mobility values.^27,28^ Operating HiKE-IMS at a low operating pressure between 10 and 40 mbar allows us to vary the reduced electric field strength *E*/*N* (the ratio of the electric field strength *E* to the number density *N*) in a wide range between 20 and 120 Td. Most notably, HiKE-IMS determines the drift time of ion species in a homogeneous electric field and thus can provide the absolute values of ion mobility depending on *E*/*N*, instead of being restricted to differential mobility values. This allows HiKE-IMS, for example, to separate HCN, H_2_S, and HCl at a high *E*/*N* of 110 Td, despite having similar low-field ion mobilities, which cannot be achieved by DTIMS at ambient pressure.^29^ Furthermore, high *E*/*N* can lead to fragmentation of analyte ions that give rise to supplementary structural information.^30−32^ In addition to its improved separation capability, the reduced number of collisions at low operating pressure mitigates chemical cross-sensitivities caused by competing ion–molecule reactions between already formed product ions and neutral interfering compounds that would otherwise lead to a charge loss of the target analyte. Due to the low number of ion–molecule collisions, the ion population is controlled by the reaction rate coefficients of the involved ion–molecule reactions rather than by the thermodynamic properties of the analytes, such as proton affinity and ionization energy. Therefore, HiKE-IMS can also reveal ions that are absent in thermodynamic equilibrium and thus expand the range of detectable compounds compared to DTIMS at ambient pressure.^33,34^ For example, HiKE-IMS can detect benzene despite its low proton affinity in the presence of toluene and xylene.^34^

Similar to HiKE-IMS, other DTIMS also operate at varying drift voltages. For instance, the voltage sweep IMS by Davis et al.^35,36^ focuses on the effect of drift voltage on resolving power, whereas the voltage sweep multiplexing IMS from Reinecke et al.^37^ aims to improve the duty cycle of IMS. Furthermore, Hauck et al.^38^ have presented an IMS for the precise measurement of ion mobility, that is operated at different *E*/*N* values up to 3 Td. Although these devices use varying drift voltages, they are all operated at ambient pressure and, thus, at low reduced field strengths. In these devices, the field-dependent effects observed in HiKE-IMS therefore have a minimal impact.

Based on the features mentioned above, we suggest HiKE-IMS as a valuable tool for trace compound detection in various applications. To further support this statement, selected CWAs, more specifically the nerve agents tabun (GA), sarin (GB), soman (GD), cyclosarin (GF), and the blister agent sulfur mustard (HD), are investigated in this work with HiKE-IMS. While HD is typically detected in negative polarity, a positive product ion has also been reported in the literature, however, only under extremely dry conditions.^39^ Due to the cluster dissociation of H_3_O^+^(H_2_O)_*n*_ at high *E*/*N* values, we expect that HiKE-IMS allows for the ionization of HD in positive polarity. To the best of our knowledge, the ionization of GB, GD, and GF in negative polarity has not been reported in the literature, but only in positive polarity. Consequently, all experiments are conducted in positive polarity, as it requires no polarity switching. In addition, common simulant agents dimethyl methylphosphonate (DMMP), diethyl methylphosphonate (DEMP), dipropylene glycol methyl ether (DPM), triethyl phosphate (TEP), and methyl salicylate (MSal) as well as potential interfering compounds such as a firefighting foam, an insecticide, and eucalyptus oil are analyzed with HiKE-IMS in positive polarity.

## Fragmentation of CWAs

Previous work has shown that product ions in HiKE-IMS are commonly subject to fragmentation, especially as the kinetic energy of ions increases due to ion heating at high *E*/*N*.^40,41^ Fragmentation pathways of CWAs have been reported in the literature, and the most relevant results for this study are summarized in Table 1. Specifically, PTR-MS at variable *E*/*N*,^42^ IMS-MS^n^,^43^ ESI-MS^n^,^44^ and GC(EI)-MS^45−47^ have been employed to examine the fragmentation patterns of CWAs. These findings are proposed to be transferable to experiments with CWAs in HiKE-IMS, and therefore, they are utilized to support the assignment of molecular formulas.

**Table 1:** Literature Findings for Fragmentation Patterns of the CWAs GA, GB, GD, GF, and HD^a^

GA (162.13 g/mol)		GB (140.09 g/mol)		GD (182.17 g/mol)		GF (180.16 g/mol)		HD (159.08 g/mol)	
molecular formula	*m*/*z*	molecular formula	*m*/*z*	molecular formula	*m*/*z*	molecular formula	*m*/*z*	molecular formula	*m*/*z*
C_3_H_8_N_2_O_2_P^+^	135^43,44^	**CH**_**5**_**FO**_**2**_**P**^**+**^	99^42−45,47^	**CH**_**5**_**FO**_**2**_**P**^**+**^	99^42−45,47^	**CH**_**5**_**FO**_**2**_**P**^**+**^	99^42−45,47^	C_3_H_6_^37^ClS^+^	111^45^
C_3_H_6_N_2_O_2_P^+^	133^45^	**CH**_**3**_**FOP**^**+**^	81^43,44^	C_6_H_13_^+^	85^43,44^	C_6_H_11_^+^	83^43^	C_3_H_6_^35^ClS^+^	109^46^
C_3_H_6_N_2_OP^+^	117^43,44^	**CH**_**4**_**O**_**2**_**P**^**+**^	79^43,44^	**CH**_**3**_**FOP**^**+**^	81^43,44^	**CH**_**3**_**FOP**^**+**^	81^43,44^		
C_2_H_7_NO_2_P^+^	108^43,44^	**PO**^**+**^	47^43^	**CH**_**4**_**O**_**2**_**P**^**+**^	79^43,44^	**CH**_**4**_**O**_**2**_**P**^**+**^	79^43,44^		
C_3_H_6_N_2_^+^	70^46^	C_3_H_7_^+^	43^43^	**PO**^**+**^	47^43^	C_4_H_7_^+^	55^43^		
				C_4_H_9_^+^	57^44^	**PO**^**+**^	47^43^		
				C_3_H_7_^+^	43^43,44^				

a Similar fragments of GB, GD and GF are highlighted in bold.

**Scheme 1:**
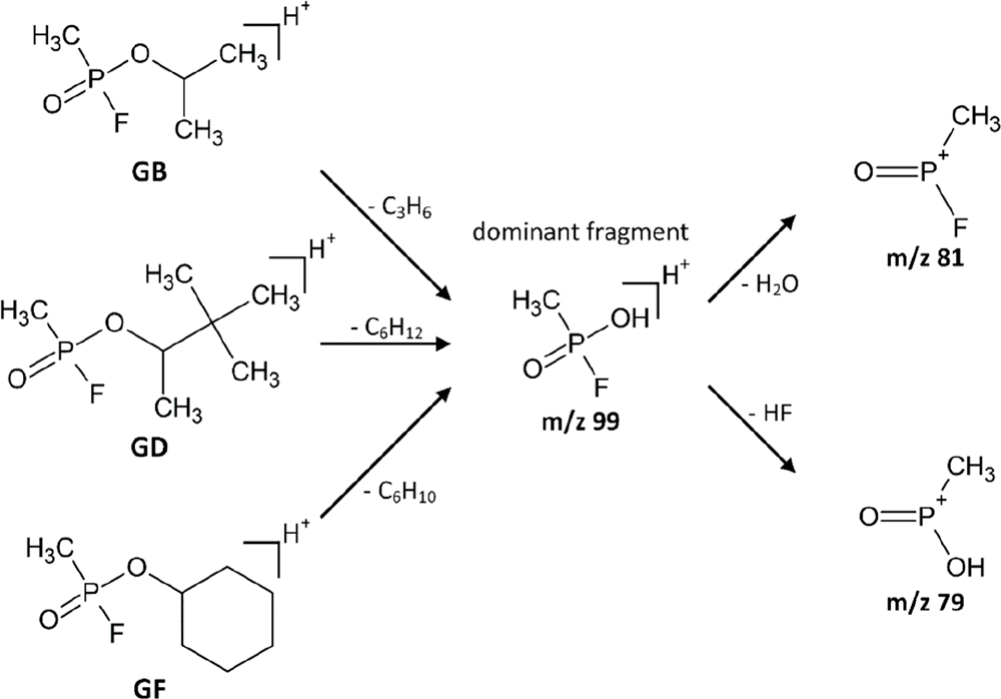
Common Fragmentation Pathway of GB, GD, and GF According to Ref (44)

The CWAs GB, GD, and GF all form *m*/*z* 99 (CH_5_FO_2_P^+^) as the dominant fragment in PTR-MS, GC-MS, IMS-MS^n^, and ESI-MS^n^, due to their structural similarity as shown in Scheme 1. Additionally, these three CWAs form further matching fragments in ESI-MS^n^ with *m*/*z* 81 (CH_3_FOP^+^) and *m*/*z* 79 (CH_4_O_2_P^+^), both resulting from dissociation of H_2_O and HF from the initial fragment CH_5_FO_2_P^+^, and *m*/*z* 47 (PO^+^) resulting from fragmentation of CH_4_O_2_P^+^. Given their similar fragmentation pathways, it is expected that GB, GD and GF generate comparable signal patterns in HiKE-IMS. In addition, both GD and GF form unique respective fragments, with *m*/*z* 85 (C_6_H_13_^+^) and *m*/*z* 57 (C_4_H_9_^+^) for GD, and *m*/*z* 83 (C_6_H_11_^+^) and *m*/*z* 55 (C_4_H_7_^+^) for GF. In contrast, the structural difference of GA results in a completely different fragmentation pattern in comparison to those of GB, GD and GF. Specifically, a fragment with *m*/*z* 135 (C_3_H_8_N_2_O_2_P^+^) is formed by loss of C_2_H_4_ from the protonated monomer. Further elimination of H_2_O or HCN leads to fragments with *m*/*z* 117 (C_3_H_6_N_2_OP^+^) and *m*/*z* 108 (C_2_H_7_NO_2_P^+^). HD forms one significant fragment in GC-MS, with the two different chlorine isotopes ^35^Cl and ^37^Cl leading to *m*/*z* 109 (C_3_H_6_^35^ClS^+^) and *m*/*z* 111 (C_3_H_6_^37^ClS^+^).

## Experimental Section

All experiments were conducted with a HiKE-IMS as described in detail in previous work.^27,48^ Briefly, HiKE-IMS uses a corona ionization source to initiate the formation of reactant ions, which then ionize neutral analyte species in a reaction region. Under dry conditions, these reactions ions are H_3_O^+^, NO^+^, and O_2_^+•^,^49^ ionizing the neutral analytes by different ionization mechanisms. Especially the ionization by charge transfer with O_2_^+•^ can lead to fragmentation of analytes,^41,50,51^ which can complicate the interpretation of the ion mobility spectra. However, previous studies have demonstrated that O_2_^+•^ is converted to H_3_O^+^(H_2_O)_*n*_ at elevated water concentrations even at high reduced field strengths.^40,52−56^ To ensure that analyte ionization primarily proceeds via proton transfer with H_3_O^+^(H_2_O)_*n*_, the relative humidity of the sample gas is intentionally increased to 50% relative humidity (rH, referred to 293 K and 1013.25 mbar), corresponding to a volume fraction of 11,500 ppm_V_. These conditions also better reflect the actual measuring environment in field applications, where the relative humidity of the sample gas can reach even higher values. Note that operation at high reduced field strengths still prevents the formation of larger hydrates of the reactant ions due to ion heating despite the high water concentration.^40,57^ A tristate ion shutter^48^ injects small ion packets into a drift region where different ion species are separated by their ion mobility. The ion current is recorded with a Faraday plate and amplified with a self-built transimpedance amplifier.^58^ The reduced field strength in the reaction region *E*_RR_/*N* and in the drift region *E*_DR_/*N* can be separately adjusted to independently control both ionization and separation in HiKE-IMS. Compared to previous setups, a self-built DC/DC converter^59^ is used for isolated power supply of electronics at high reference potential, e.g. for the control electronics of the tristate ion shutter and the power supplies of the corona ionization source and the reaction region. Table 2 summarizes the most relevant operating parameters of HiKE-IMS used in this work.

**Table 2:** Operating Parameters of the HiKE-IMS

Parameter	Value
Reaction region length	75 mm
Drift region length	231 mm
Corona voltage	1200 V
Reaction region voltage	600–2800 V
Drift region voltage	1800–8500 V
Reduced reaction field strength *E*_RR_/*N* and reduced drift field strength *E*_DR_/*N*	25–120 Td
Injection time	1 μs
Drift gas flow rate^a^	18 mL/min
Sample gas flow rate^a^	36 mL/min
Operating pressure	13–14 mbar
Temperature HiKE-IMS *T*_IMS_	50 °C
Temperature sample inlet *T*_Inlet_	40 °C

a Gas flow rates correspond to reference conditions 293 K and 1013.25 hPa.

### Chemicals and Gas Supply

The nerve agents GA, GB, GD, and GF and the blister agent HD were synthesized at the Bundeswehr Research Institute for Protective Technologies and CBRN Protection (WIS). The purities were determined by quantitative nuclear magnetic resonance (NMR) spectroscopy and were 94.0% for GA, 93.3% for GB, 92.4% for GD, 89.1% for GF and 99.7% for HD. The simulant agents DMMP, DEMP, DPM, TEP and MSal were purchased from Sigma-Aldrich with purities >97.5%. As potential interfering compounds, the firefighting foam “Aqueous Film Forming Foam (AFFF)” by 3M, the insecticide “Anti Brumm” by Hermes Arzneimittel GmbH (active substance diethyltoluamide (DEET)) and the eucalyptus oil “Pflanzliche Erkältungstropfen” by Optisana were used.

**Figure 1:**
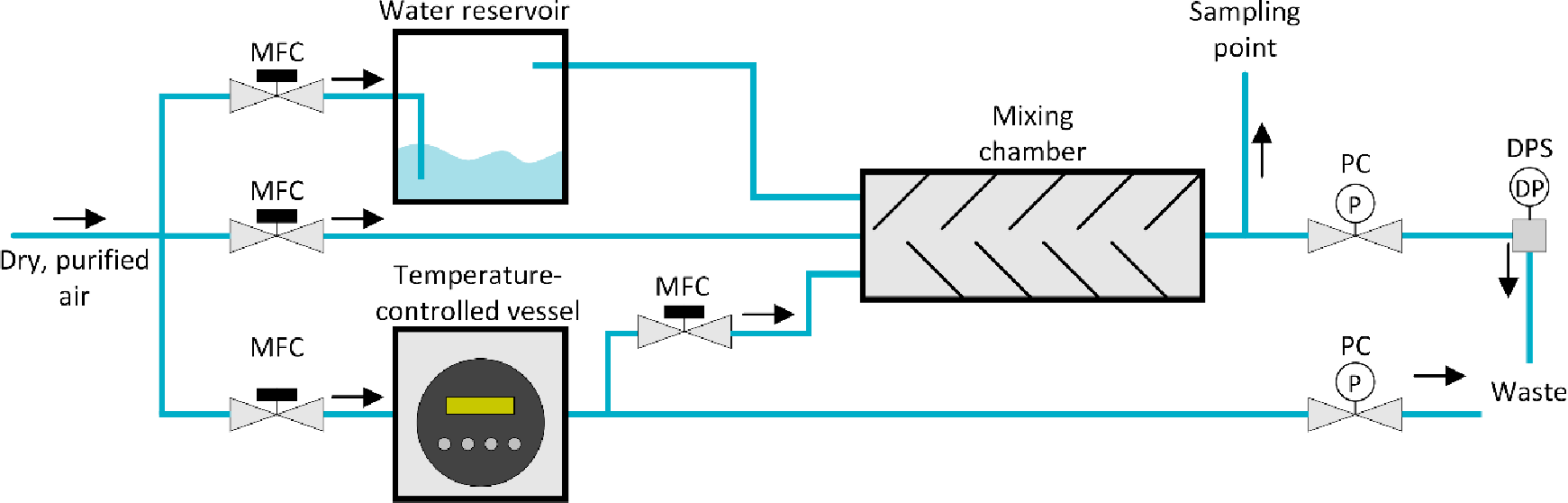
Gas mixing system for sample gas preparation using mass flow controllers (MFC), pressure controllers (PC), a dew point sensor (DPS), a temperature-controlled vessel, a water reservoir, and a gas mixing chamber.

For sample gas preparation, the setup shown in Figure 1 was used. It consists of mass flow controllers (MFC), pressure controllers (PC), a temperature-controlled vessel, a water reservoir, and a gas mixing chamber. The gas mixing chamber can be fed by dry air, humidified air, and air from a temperature-vessel containing a permeation tube filled with one of the above-mentioned CWAs. The composition of the gas at the sampling point downstream of the gas mixing chamber can be controlled by varying the mixing ratios. The moisture content of the gas is additionally monitored with a dew point sensor (DPS). The test gas concentration was determined by loading Tenax TA adsorption tubes downstream of the mixing chamber and subsequent analysis by thermal desorption on a GC-MS. For calibration, Tenax TA tubes were spiked with appropriate methanolic solutions containing 5, 10, 20, 30, 40, and 50 ng of sample reference. CWAs were solely handled at WIS laboratories, applying stringent safety procedures.

### Method

Previous studies of product ion formation in HiKE-IMS have shown that *E*_RR_/*N* significantly affects the ion chemistry by controlling the reactant ion population and by causing field-induced fragmentation of the product ions.^41,60,61^ For some analytes, choosing a high value for *E*_RR_/*N* may result in the formation of fragments only, with loss of specific structural information about the parent ion species.^61^ However, since the overall signal intensity typically increases with increasing *E*_RR_/*N*,^61,62^ analyte-specific optimization of *E*_RR_/*N* is recommended. Therefore, to study the individual ion formation of the examined CWAs with regard to the reduced electric field strength, *E*_RR_/*N* is varied between 25 and 120 Td in steps of 1 Td, respectively. For *E*_DR_/*N*, a comparatively low value of *E*_DR_/*N* = 25 Td is chosen to avoid additional fragmentation within the drift region. In a next experimental series, *E*_DR_/*N* is varied between 25 and 120 Td in steps of 1 Td, respectively, to evaluate the field dependence of ion mobility as an additional separation dimension. Again, to avoid significant fragmentation of the parent ions in the reaction region, a comparatively low value of *E*_RR_/*N* = 25 Td was chosen in this experiment. By independently varying the reduced field strength in the reaction region and the drift region, the influence of *E*/*N* on ion formation, fragmentation, and separation can be investigated individually. Furthermore, the experiments of this study can be used to evaluate whether the variation of *E*_RR_/*N* or *E*_DR_/*N* is more useful for reliable detection of CWAs with HiKE-IMS. Note that the selected step size affects both the analysis time and the precision of additional information provided by HiKE-IMS. In this study, we have chosen a small step size and averaged each ion mobility spectrum 32,000 times. While this approach is extremely time-consuming, requiring approximately 150 min, it allows for a very detailed investigation of ion formation and ion mobility. In practical applications, neither the high number of averages per ion mobility spectrum, nor the small step size is necessary. Consequently, analysis times of only a few seconds are possible. With the limits of detection given for 2 s of averaging at a fixed *E*/*N* determined below, the overall analysis time can be calculated as the number of *E*/*N* values multiplied with the 2 s of averaging. E.g., analyzing at one low, one mid, and one high *E*/*N*, the total analysis time sums up to 6 s while reaching the limits of detection stated below. Changing the *E*/*N* value only needs milliseconds and is therefore negligible.

## Results and Discussion

### Effect of Reduced Reaction Field Strength *E*_RR_*/N*

**Figure 2:**
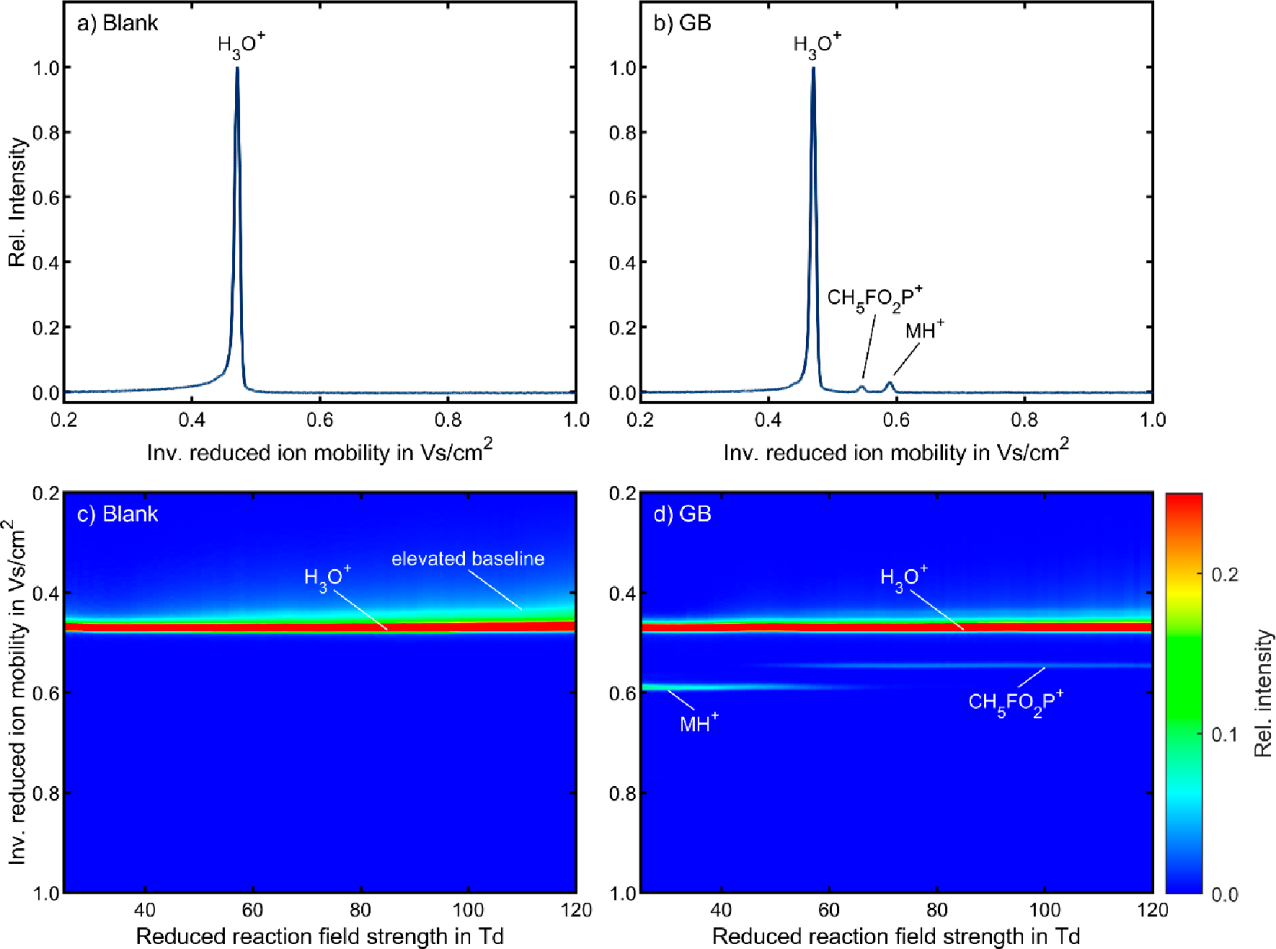
Comparison between a blank measurement and GB in HiKE-IMS in positive polarity in air with relative humidity of 50% and at *E*_DR_/*N* = 25 Td: Ion mobility spectrum of (a) a blank measurement and (b) 218 ppb_V_ GB, each at E_RR_/*N* = 55 Td. Topographic plots with variation of the reduced reaction field strength for (c) a blank measurement and (d) 218 ppb_V_ GB. Each individual ion mobility spectrum is normalized to its maximum value of intensity. Each topographic plot is scaled to 25% of the maximum value for better visibility. All other operating parameters were set according to Table 2.

We started our investigation by studying the effect of the reduced reaction electric field strength, *E*_RR_/*N* in HiKE-IMS on the chosen CWAs. As a point of reference, we always conducted a blank measurement without any analytes prior to each experimental series. Figure 2 shows a comparison of exemplary ion mobility spectra of a blank measurement (a) and GB (b), each recorded at *E*_DR_/*N* = 25 Td and *E*_RR_/*N* = 55 Td. The blank measurement (a) reveals only one significant ion species, which has been identified in previous work as H_3_O^+^(H_2_O)_*n*_.^40,52,28^ The corresponding topographic plot in Figure 2c shows the influence of *E*_RR_/*N* on the ion mobility spectra. The topographic plot shows an elevated baseline as fronting of the peak of H_3_O^+^(H_2_O)_*n*_, especially at high *E*_RR_/*N*. At low *E*_RR_/*N*, NO^+^, another relevant reactant ion in HiKE-IMS, can form a hydrate with three water molecules NO^+^(H_2_O)_3_, which reacts with water to eventually form hydrated hydronium ions.^49,63^ This conversion reaction is inhibited at high *E*_RR_/*N*, because the hydrates of the reactant ions dissociate at high kinetic energies.^60^ Correspondingly, NO^+^(H_2_O)_*m*_ is injected into the drift region at a higher *E*_RR_/*N*. The injected NO^+^(H_2_O)_*m*_ can still form the unstable trihydrate in the drift region at the low reduced drift field strength of 25 Td, which is then converted to hydrated hydronium ions. Since this conversion reaction takes place within the drift region, the observed ion mobility for these ions is a weighted average of the ion mobilities of NO^+^(H_2_O)_*m*_ and H_3_O^+^(H_2_O)_*n*_, leading to the observed fronting of the H_3_O^+^(H_2_O)_*n*_ peak. The monohydrate of O_2_^+•^ is also converted to hydrated hydronium ions even at *E*_RR_/*N* = 120 Td due to the high relative humidity of 50%.^60,49^ This ensures that analyte ionization proceeds primarily by proton transfer with H_3_O^+^(H_2_O)_*n*_.

As expected, the ion mobility spectrum of GB shows a number of characteristic signals (Figure 2b). The ion species with a reduced ion mobility of *K*_0_ = 1.695 cm^2^/(V s) has been identified as the protonated monomer (MH^+^) based on the good agreement with the literature value for the reduced ion mobility of MH^+^ (*K*_0_ = 1.688 cm^2^/(V s)).^5^ A second product ion peak with a higher reduced ion mobility of *K*_0_ = 1.832 cm^2^/(V s) is attributed to fragment CH_5_FO_2_P^+^ (*m*/*z* 99). Due to the high kinetic energies of the ions at high *E*_RR_/*N* in HiKE-IMS, we expect a fragmentation pathway similar to the literature as shown in Scheme 1. The topographic plot in Figure 2d shows that the relative intensity of the product ions decreases constantly with increasing *E*_RR_/*N*. This can be ascribed to the decreasing residence time of the reactant ions in the reaction region since their drift velocity increases at high E_RR_/N. Consequently, the decreasing reaction time leads to a decrease in the relative intensity of the product ions. It is also noteworthy that the relative intensity of the protonated monomer decreases between 50 and 60 Td as a result of its fragmentation. Correspondingly, the relative intensity of the generated fragment shows a major increase in this E_RR_/*N* range.

Ion mobility spectra of other selected CWAs obtained under the same operating conditions as well as the corresponding topographic plots with variation of *E*_RR_/*N* can be found in Supporting Information in Section S1. Ion mobilities of all observed product ion species at *E*_DR_/*N* = 25 Td are summarized in Table 3 in the following section. The good agreement of the reduced ion mobilities obtained by HiKE-IMS operating below 50 Td with literature values obtained by IMS operating at ambient pressure suggests that the protonated monomers of GA (*K*_0_ = 1.563 cm^2^/(V s)), GD (*K*_0_ = 1.486 cm^2^/(V s)), and GF (*K*_0_ = 1.484 cm^2^/(V s)) are present in our experiments.^5^ HD yields one major ion species in positive polarity with a reduced ion mobility of *K*_0_ = 1.672 cm^2^/(V s). As discussed above, although HD is typically detected in negative polarity, a product ion in positive polarity has been reported in literature.^39^ The literature value for the most abundant product ion found by Sohn and Steinhanses^39^ of *K*_0_ = 1.66 cm^2^/(V s) in positive polarity agrees well with the ion mobility determined in HiKE-IMS at *E*_DR_/*N* = 25 Td. Interestingly, this ion species can only be detected above *E*_RR_/*N* = 50 Td in HiKE-IMS. As shown by Sohn and Steinhanses, HD can only be ionized at dry conditions, when small hydrates of H_3_O^+^ are present. Due to the presence of larger hydrates of H_3_O^+^ at low *E*_RR_/*N*, the ionization of HD is inhibited under these conditions. Contrarily, at higher *E*_RR_/*N*, the cluster dissociation of the hydrated hydronium ions enables the ionization of HD by proton transfer.

**Table 3:** Determined Reduced Ion Mobilities of the Investigated CWAs, Simulant Agents and Potential Interfering Compounds at Different Reduced Drift Field Strengths in Air at *E*_RR_/*N* = 25 Td in Comparison with the Literature Values of the Low-Field Ion Mobility from Ref (5)^a^

		*K*_0_ in cm^2^/(V s) at *E*_DR_/*N* = 25 Td	*K*_0_ in cm^2^/(V s) at *E*_DR_/*N* = 60 Td	*K*_0_ in cm^2^/(V s) at *E*_DR_/*N* = 110 Td	Low-field ion mobility in cm^2^/(V s) according to ref ^5^
GA	fragment	1.597	1.663	1.719	NA
	MH^+^	1.563	1.606	1.639	1.563
GB	CH_5_FO_2_P^+^	1.832	1.891	1.998	NA
	MH^+^	1.695	1.742	NA	1.688
GD	fragment 2	2.011	2.037	2.058	NA
	fragment 3	1.925	NA	NA	NA
	CH_5_FO_2_P^+^	1.832	1.891	1.998	NA
	MH^+^	1.486	NA	NA	1.486
GF	CH_5_FO_2_P^+^	1.832	1.891	1.998	NA
	MH^+^	1.484	1.505	NA	1.490
HD	MH^+^	1.672	1.687	1.696	1.66^39^
DMMP	peak 1	1.836	1.906	1.938	1.804
	peak 2	1.423	1.416	1.389	1.410
DEMP	peak 1	1.677	1.719	1.727	1.648
	peak 2	1.235	1.228	1.205	1.220
DPM	peak 1	1.686	1.705	1.706	1.653
	peak 2	1.237	1.229	1.210	1.223
TEP	peak 1	1.577	1.612	1.609	1.559
	peak 2	1.131	1.127	1.106	1.230
firefighting foam	peak 1	1.732	1.750	1.751	NA
	peak 2	1.565	1.585	1.583	1.534
insecticide	peak 1	1.472	1.476	1.465	NA
eucalyptus oil	peak 1	NA	NA	1.693	NA
	peak 2	1.579	1.605	NA	1.555
methyl salicylate	peak 1	1.735	1.751	1.755	1.709

a The literature value for HD in positive polarity was taken from Ref (39).

**Figure 3:**
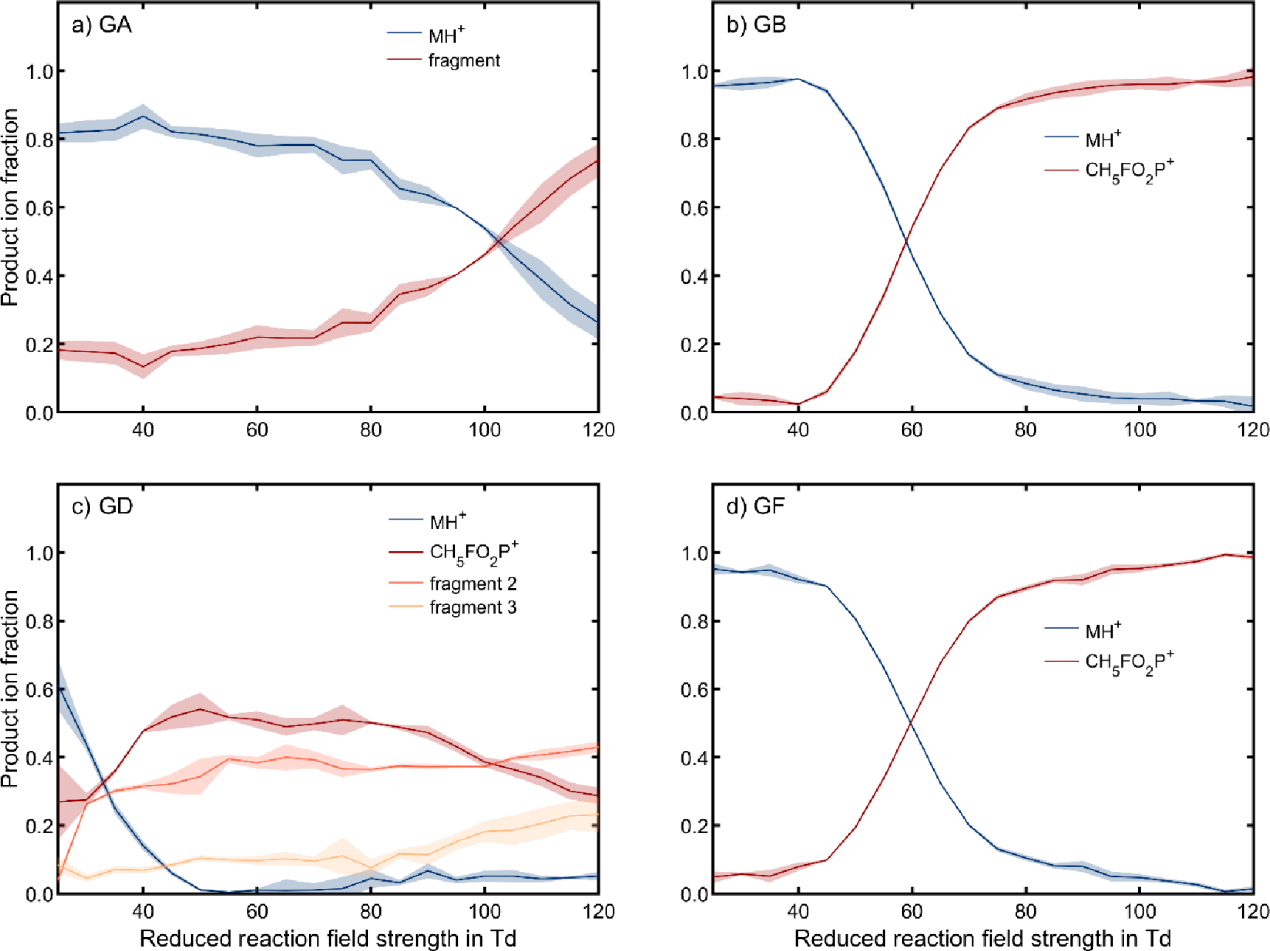
Product ion populations of CWAs (a) 252 ppb_V_ GA, (b) 218 ppb_V_ GB, (c) 247 ppb_V_ GD, and (d) 244 ppb_V_ GF in positive polarity in air with relative humidity of 50% and at *E*_DR_/*N* = 25 Td depending on *E*_RR_/*N*. Product ion fractions are determined by normalizing the peak area of each individual product ion to the sum of peak areas of all product ions. All other operating parameters were set according to Table 2. The error bands show the standard deviations of three individual measurements.

Figure 3 shows the peak area of each product ion normalized to the sum of peak areas of all product ions for each CWA, i.e. the product ion fractions, depending on *E*_RR_/*N*. The graphs provide an improved illustration of the extent of product ion fragmentation by mitigating the effect of varying reaction times. HD is absent in Figure 3 because it shows no visible fragments in this experiment and thus has only one significant product ion species. As *E*_RR_/*N* increases, the relative abundance of the respective protonated monomer of GA, GB, GD and GF decreases. Correspondingly, the relative abundances of ion species with higher ion mobilities increase, indicating fragmentation within the reaction region. While CWAs GB, GD and GF form protonated molecular ions as specific signals at low *E*_RR_/*N*, they all form a common fragment at high *E*_RR_/*N*. This indicates a similar fragmentation pathway for these CWAs, which is unsurprising given their structural similarity. As discussed above, these derivatives also form the same dominant fragment in PTR-MS, GC-MS, IMS-MS^n^ and ESI-MS^n^. As with GB, we suggest that this common fragment is attributed to the ion species CH_5_FO_2_P^+^ (*m*/*z* 99) with *K*_0_ = 1.832 cm^2^/(V s). GD forms two additional fragments at high *E*_RR_/*N* with even higher reduced ion mobilities (*K*_0_ = 1.925 cm^2^/(V s) and *K*_0_ = 2.011 cm^2^/(V s)) than CH_5_FO_2_P^+^, which we have been unable to elucidate as yet. The formation of different product ions at varying *E*_RR_/*N* generally provides additional information about the analyte that may help to reduce false positive alarms caused by interfering compounds. Importantly, however, the reduced reaction field strength must be chosen carefully to preserve a distinction between certain CWAs due to similar fragmentation patterns of structurally related derivatives at high *E*_RR_/*N*. GA appears to be less prone to fragmentation in comparison to GB, GD and GF: its protonated monomer starts noticeable fragmentation only above 100 Td and is still observable at 120 Td. The generated fragment has a higher reduced ion mobility of *K*_0_ = 1.597 cm^2^/(V s) than that of the protonated monomer of *K*_0_ = 1.563 cm^2^/(V s).

### Effect of Reduced Drift Field Strength *E*_DR_*/N*

**Figure 4:**
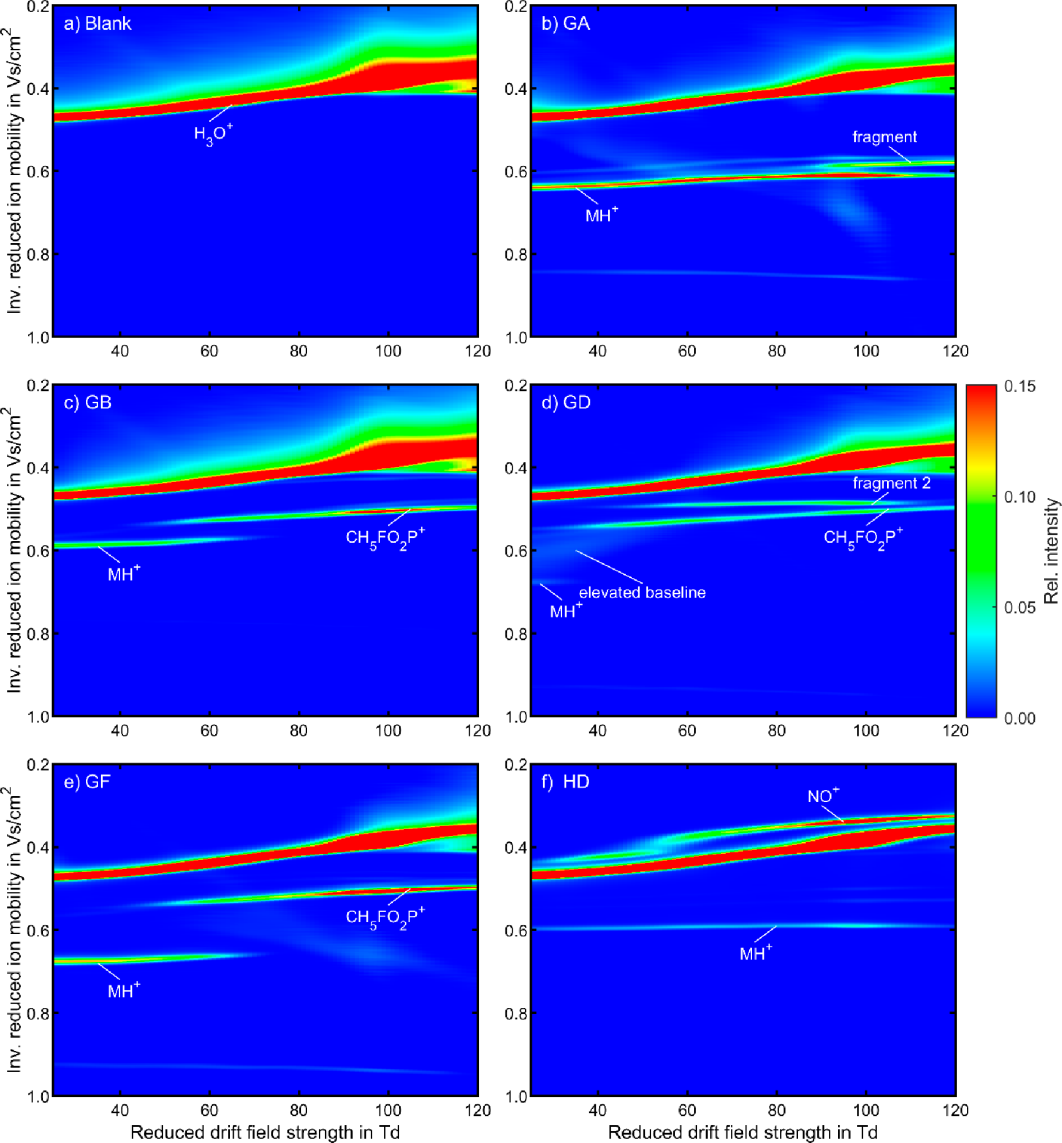
Dispersion plots of (a) a blank measurement in comparison to the chemical warfare agents (b) 252 ppb_V_ GA, (c) 218 ppb_V_ GB, (d) 247 ppb_V_ GD, and (e) 244 ppb_V_ GF at E_RR_/*N* = 25 Td and of f) 245 ppb_V_ HD at E_RR_/*N* = 75 Td in positive polarity in air with relative humidity of 50%. Each individual ion mobility spectrum is normalized to its maximum value of intensity. Each dispersion plot is scaled to 15% of the maximum value for better visibility. All other operating parameters were set according to Table 2.

We investigated the effect of *E*_DR_/*N* on both the ion population and the field dependence of the ion mobility at a constant reduced reaction field strength of *E*_RR_/*N* = 25 Td. The resulting dispersion plots, visualizing the field dependence of the ion mobility of all investigated CWAs, including a blank measurement, are shown in Figure 4. Table 3 summarizes the extracted ion mobilities at 25, 60, and 110 Td. As expected, the reactant ion species H_3_O^+^(H_2_O)_*n*_ is the only significant ion species in the blank measurement. The elevated baseline as fronting of the peak of H_3_O^+^(H_2_O)_*n*_ is caused by NO^+^(H_2_O)_*m*_ (*vide supra*). The field dependence of the ion mobility for the hydrated hydronium ions has been discussed in detail in previous work.^28^

Similar to prior experiments with varying *E*_RR_/*N*, as described in the previous section, each CWA forms its protonated monomer at low *E*_DR_/*N* < 30 Td with the same ion mobility as in the previous experiments. GA, GB, GD and GF show additional signals from fragmentation at increased *E*_DR_/*N* with higher ion mobilities according to Table 3. Similar to the findings in the previous section, the signal intensities of the fragments increase with increasing *E*_DR_/*N*. The protonated monomer is absent at higher *E*_DR_/*N* values for GB, GD and GF. Instead, the common fragment CH_5_FO_2_P^+^ can be found for all of these CWAs, with a reduced ion mobility of *K*_0_ = 1.891 cm^2^/(V s) at *E*_DR_/*N* = 60 Td. The perceptible fragmentation of GB and GF starts at about *E*_DR_/*N* > 40 Td, whereas GD already starts fragmenting at 25 Td. The elevated baseline between the protonated monomer of GD and CH_5_FO_2_P^+^ is possibly a result of fragmentation of the protonated monomer in the drift region. In case of a fragmentation of the ion within the drift region, the observed CH_5_FO_2_P^+^ ion mobility is an average of the individual ion mobilities weighted by the fraction of the drift time that the ion drifts as one of the two species.^64,65^ The statistical distribution of the position of fragmentation results in an elevated baseline observed in the ion mobility spectrum. As *E*_DR_/*N* increases, increasing the kinetic energy leads to faster fragmentation, with no significant peak broadening or elevated baseline observed. This indicates that at high *E*_DR_/*N* the fragmentation within the drift region is fast compared to the drift time. The other investigated CWAs show no elevated baseline in the corresponding dispersion plots, indicating faster fragmentation in the drift region. Note that, besides fragmentation, other reactions in the drift region, especially cluster dissociation due to ion heating at higher reduced drift field strengths, could also cause the observed peak shape and a change in the reduced ion mobility. In contrast, the protonated monomer of GA appears to be less prone to fragmentation, confirming the earlier findings from variation of *E*_RR_/*N*. While a fragment is observed above *E*_DR_/*N* = 90 Td, the protonated monomer is still visible even at 120 Td. The dispersion plot for HD was recorded at *E*_RR_/*N* = 75 Td, since its product ions are exclusively formed at higher *E*_RR_/*N*. Nevertheless, HD shows its protonated monomer as the only signal, even at a high *E*_DR_/*N*. Note that the reactant ion NO^+^ is also observed as a distinct signal at *E*_RR_/*N* = 75 Td, since its conversion to hydrated hydronium ions is inhibited by cluster dissociation.^49^

In all experiments, the protonated monomers and fragments show an increase in ion mobility with increasing *E*_DR_/*N*. This effect may be explained by the dynamic clustering behavior of these ions.^66,67^ Comparatively small ions may form hydrates at low *E*_DR_/*N* values that dissociate at high *E*_DR_/*N*. Thus, the observed change in ion mobility is proposed to be a result of a decrease in collision cross sections with an increasing *E*_DR_/*N*. Furthermore, the dispersion plots in Figure 4 show that all five investigated CWAs are clearly distinguished from each other. Each agent has its unique fingerprint encompassing the field dependence of ion mobility, fragmentation at high *E*_DR_/*N* and specific signals given by the protonated monomers at low *E*_DR_/*N*. Most notably, the additional information provided by fragmentation at higher *E*_DR_/*N* permits a distinct differentiation between GD and GF, which have almost identical low-field ion mobilities according to our experiments and the literature.^5,8,10^ The unique fingerprint can also reduce false positives since interfering compounds are less likely to have a matching low-field ion mobility together with the same field dependence of the ion mobility as well as a comparable formation of fragments at varying reduced field strength. To investigate the effect of interfering compounds on CWA detection with HiKE-IMS, dispersion plots of the simulants DMMP, DEMP, DPM, TEP and methyl salicylate as well as those of the interferents firefighting foam, insect spray, and eucalyptus oil are recorded for *E*_DR_/*N* between 25 and 110 Td in steps of 5 Td at *E*_RR_/*N* = 25 Td and shown in the Supporting Information (Figures S3 and S4). According to Table 3, the reduced ion mobilities of the dominant signals of the interfering compounds at low *E*_DR_/*N* are in a range similar to those of the CWAs. In particular, the firefighting foam (*K*_0_ = 1.565 cm^2^/(V s)), eucalyptus oil (*K*_0_ = 1.579 cm^2^/(V s)) and the protonated monomer of GA (*K*_0_ = 1.563 cm^2^/(V s)) have similar reduced ion mobilities at *E*_DR_/*N* = 25 Td. However, as *E*_DR_/*N* is varied, these can be clearly distinguished due to different field dependencies of the ion mobility. Eucalyptus oil also forms a fragment at high *E*_DR_/*N* with *K*_0_ = 1.693 cm^2^/(V s) at 110 Td, but the fragmentation pattern can be clearly distinguished from those of CWAs due to the different ion mobilities of the individual fragments and fragmentation occurring at different *E*_DR_/*N* values.

In conclusion, the experiments with selected CWAs show that variation of both *E*_RR_/*N* and *E*_DR_/*N* can lead to a higher degree of fragmentation with increasing *E*/*N*, while specific signals such as the protonated monomers are present at lower *E*/*N* values. The additional information contributes to a more reliable detection of CWAs. Moreover, when varying *E*_DR_/*N*, the field dependence of the ion mobility is available as another separation dimension in addition to the ion mobility at a single reduced field strength, thus, increasing detection reliability even further. As a result, variation of *E*_DR_/*N* is considered the most promising approach when HiKE-IMS is used for more reliable detection of CWAs.

### Sensitivity of HiKE-IMS

**Figure 5:**
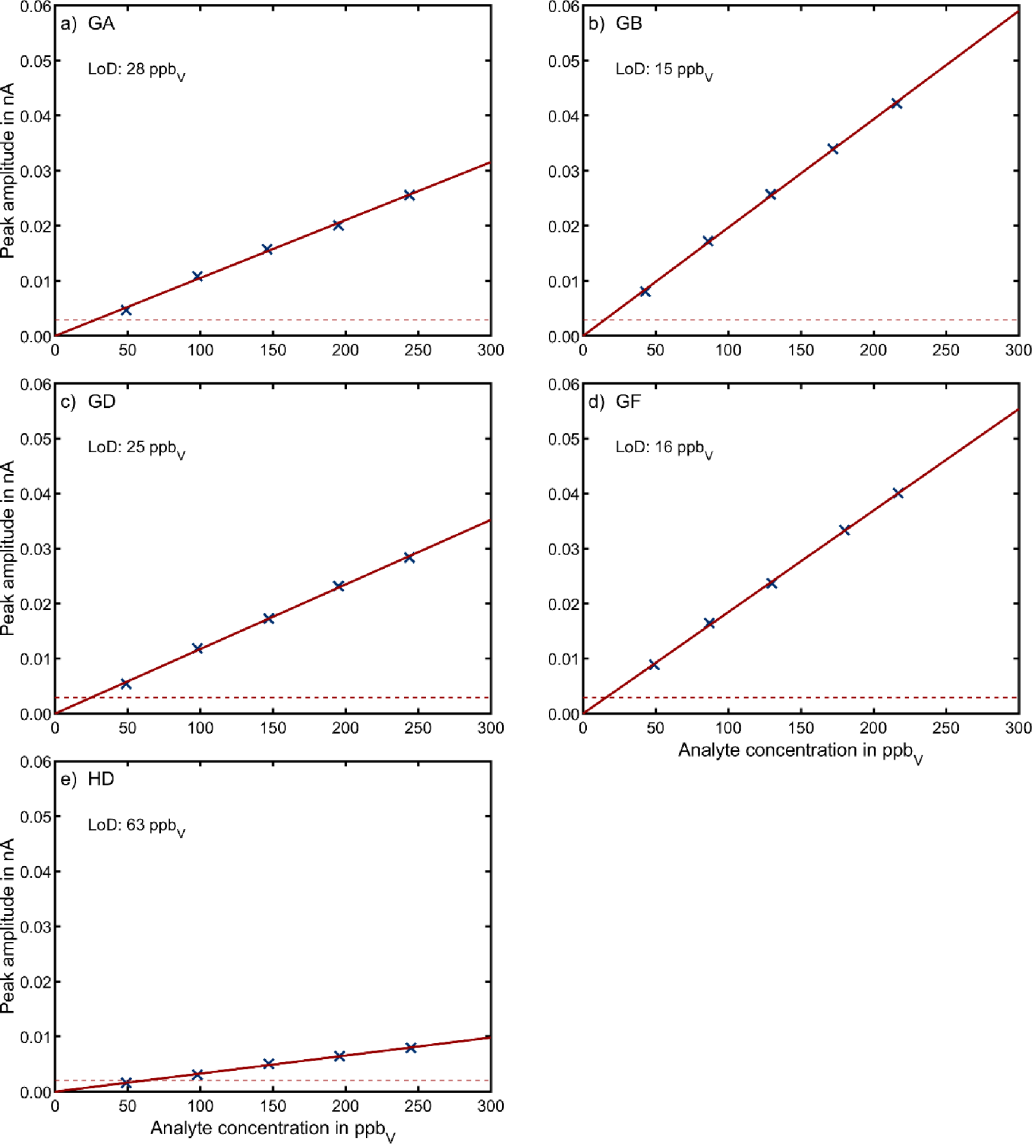
Calibration curves for a) GA, b) GB, c) GD, d) GF, and e) HD at E_RR_/*N* = E_DR_/*N* = 120 Td in air with relative humidity of 50%. The peak amplitudes were determined for the peaks highlighted in the ion mobility spectra in Figure S5. The solid red line represents a linear fit of the peak amplitudes to the vapor concentration. Limits of detection (LoD) were determined by dividing three times the standard deviation at c = 0 ppb_V_ (blank measurement) obtained for an averaging time of 2 s (marked as dashed lines in the calibration curves) by the slope of the linear fit. All other operating parameters were set according to Table 2.

Ion mobility spectra of CWAs were recorded at different vapor concentrations at constant reduced field strengths of *E*_RR_/*N* = *E*_DR_/*N* = 120 Td. The obtained peak amplitudes were used to evaluate the sensitivity of HiKE-IMS for each agent. Example ion mobility spectra are shown in Figure S5, highlighting the peaks used to determine the calibration curves shown in Figure 5. The respective limit of detection (LoD) was determined by dividing three times the standard deviation of a blank measurement, obtained with an averaging time of 2 s, by the slope of the linear fit that was applied to the calibration data points.

The calibration curves provide a linear dependence of the signal intensity on the vapor concentration within the investigated concentration range. The results highlight the major drawback of HiKE-IMS, which is the lower sensitivity compared to IMS operating at ambient pressure.^5^ Still, the limit of detection for an analysis time of 2 s is below the 10 min marginal military exposure guidelines (MEG)^68^ for GB and HD, as shown in Table 4. We stress that the HiKE-IMS used in this study has yet to be optimized for sensitivity. In this context, previous work has already shown that increasing the operating pressure of HiKE-IMS to 40 or 60 mbar improves the sensitivity of HiKE-IMS while still allowing for high *E*_RR_/*N* and *E*_DR_/*N*.^33,69^ Nevertheless, by switching HiKE-IMS between only a few *E*/*N* values, as discussed above, the analysis time of 2 s per individual ion mobility spectrum allows users in practical applications to benefit from the additional information provided by HiKE-IMS for more reliable detection still within a few seconds.

**Table 4:** Limits of Detection of HiKE-IMS for CWAs Investigated in This Work in Comparison to the 10 min Marginal and 10 min Negligible MEGs^68^

agent	LoD (this work)	MEG 10 min marginal	MEG 10 min negligible
GA	28 ppb_V_ (185.6 μg/m^3^)	140 μg/m^3^	6.9 μg/m^3^
GB	15 ppb_V_ (85.9 μg/m^3^)	140 μg/m^3^	6.9 μg/m^3^
GD	25 ppb_V_ (186.2 μg/m^3^)	61 μg/m^3^	3.5 μg/m^3^
GF	16 ppb_V_ (117.8 μg/m^3^)	57 μg/m^3^	3.5 μg/m^3^
HD	63 ppb_V_ (409.7 μg/m^3^)	1200 μg/m^3^	400 μg/m^3^

## Conclusion

In this work, we analyzed the CWAs GA, GB, GD, GF, and HD using HiKE-IMS with a focus on ion formation, field-induced fragmentation, and field-dependent ion mobility. The results show that GB, GD, and GF form a common fragment at high *E*/*N*, while GA shows little fragmentation and HD forms no observable fragments. Combined with the specific information given by signals of the respective protonated monomers at low *E*/*N*, the fragmentation pattern allows for improved identification of CWAs. In comparison to the variation of *E*_RR_/*N* at constant *E*_DR_/*N*, the variation of *E*_DR_/*N* at constant *E*_RR_*/N* has shown to further increase reliability of identification since it combines ion separation under low field conditions with ion separation using the field-dependent ion mobility. Thus, varying *E*_DR_/*N* creates a unique fingerprint for each analyte. This potentially reduces false positives and allows for more reliable detection of CWAs while facilitating the differentiation of CWAs from possible interfering compounds. These benefits make HiKE-IMS a powerful tool for trace compound analysis. Further work will focus on improving the sensitivity of HiKE-IMS.

## Supporting Information Available

Topographic plots of blank measurements and CWAs with variation of the reduced reaction field strength *E*_RR_/*N* at *E*_DR_/*N* = 25 Td; ion mobility spectra of blank measurements and CWAs at *E*_RR_/*N* = 25 Td and *E*_DR_/*N* = 55 Td; dispersion plots of simulants; dispersion plots of interfering compounds; ion mobility spectra of blank measurements and CWAs at *E*_RR_/*N* = 120 Td and *E*_DR_/*N* = 120 Td (PDF)
