# Supplementary material for: Reliable Detection of Chemical Warfare Agents Using High Kinetic Energy Ion Mobility Spectrometry
Source: J Am Soc Mass Spectrom. 2024 Jul 16;35(8):2008–19. doi: 10.1021/jasms.4c00240 (PMC11311216; doi:10.1021/jasms.4c00240)
Supplement: Supplementary file 1 — js4c00240_si_001.pdf [file js4c00240_si_001.pdf]

Supporting Information

Reliable Detection of Chemical Warfare Agents Using High Kinetic Energy Ion Mobility Spectrometry

Christoph Schaefer[1*], Maria Allers[2*], Moritz Hitzemann[1], Alexander Nitschke[1], Tim Kobelt[1], Max Mörtel[2], Stefanie Schröder[2], Arne Ficks[2], Stefan Zimmermann[1]

[1] Leibniz University Hannover, Institute of Electrical Engineering and Measurement Technology, Department of Sensors and Measurement Technology, Appelstr. 9A, 30167 Hannover, Germany

[2] Bundeswehr Research Institute for Protective Technologies and CBRN Protection, Munster 29633, Germany

*Corresponding Authors: schaefer@geml.uni-hannover.de; mariallers@bundeswehr.org

Table of contents

Figure S1. Heatmaps of GA, GB, GD and HD depending on E_{RR}/N in positive polarity in air with relative humidity of 50 % and at E_{DR}/N = 25 Td.. 2
Figure S2. Ion mobility spectra of GA, GB, GF and HD in positive polarity in air with relative humidity of 50 % and at E_{RR}/N = 25 Td and E_{DR}/N = 55 Td. 3
Figure S3. Dispersion plots of simulants DMMP, DEMP, DPM and TEP in positive polarity in dry air at E_{RR}/N = 25 Td 4
Figure S4. Dispersion plots of interferents AFFF, insecticide, eucalyptus oil and the simulant methyl salicylate in positive polarity in dry air at E_{RR}/N = 25 Td...5
Figure S5. Ion mobility spectra of a blank measurement in comparison to chemical warfare agents in positive polarity in air with relative humidity of 50 % and at E_{RR}/N = E_{DR}/N = 120 Td 6

Section S1. Heatmaps and Ion Mobility Spectra of CWAs

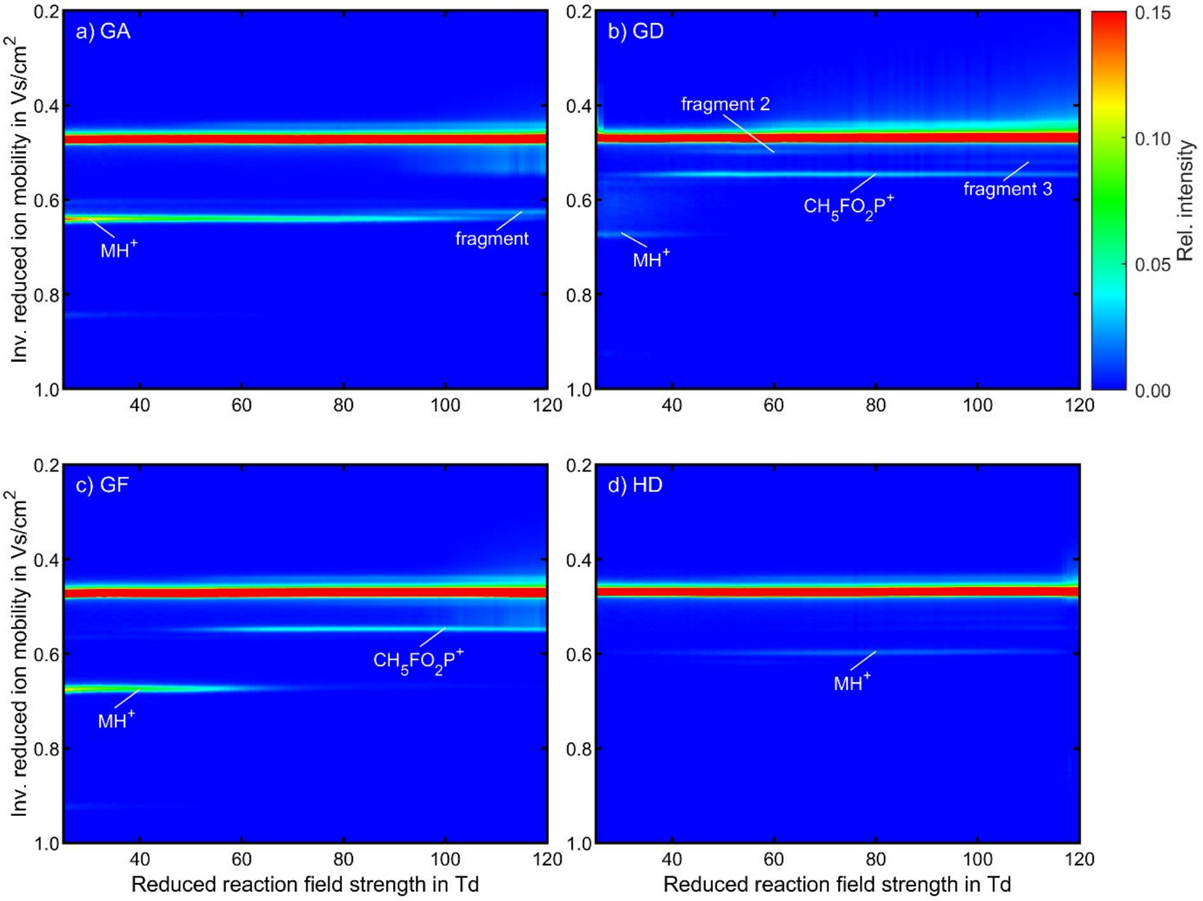

Figure S1. Topographic plots of a) 244 ppb$_V$ GA, b) 247 ppb$_V$ GD, c) 233 ppb$_V$ GF and d) 245 ppb$_V$ HD depending on E_{RR}/N in positive polarity in air with relative humidity of 50 % and at E_{DR}/N = 25 Td. Each individual ion mobility spectrum is normalized to its maximum value of intensity. Each topographic plot is scaled to 15 % of the maximum value for better visibility. All other operating parameters were set according to Table 1 in the main manuscript.

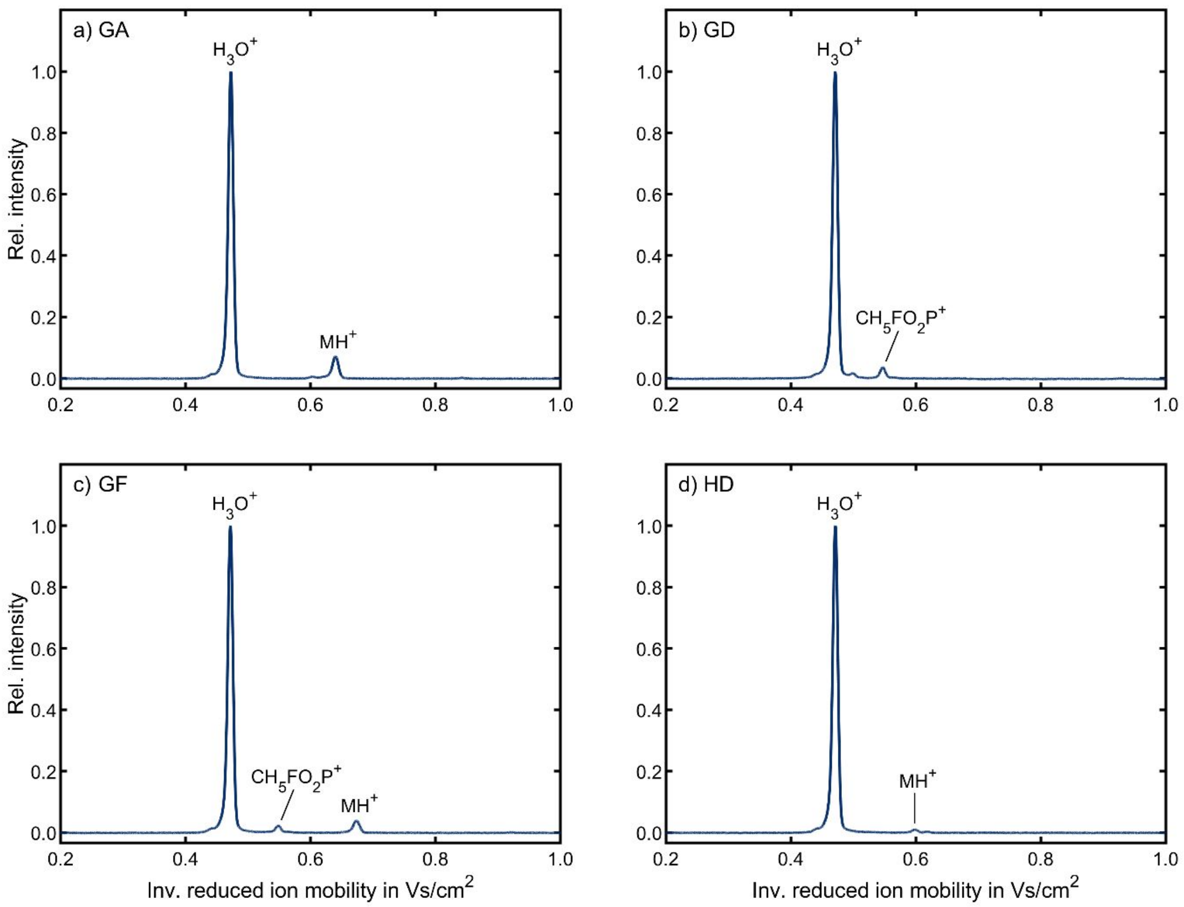

Figure S2. Ion mobility spectra of a) 244 ppb$_V$ GA, b) 247 ppb$_V$ GD, c) 233 ppb$_V$ GF and d) 245 ppb$_V$ HD in positive polarity in air with relative humidity of 50 % and at E_{RR}/N = 25 Td and E_{DR}/N = 55 Td. All other operating parameters were set according to Table 1 in the main manuscript.

Section S2. Dispersion Plots of Simulants and Interferents

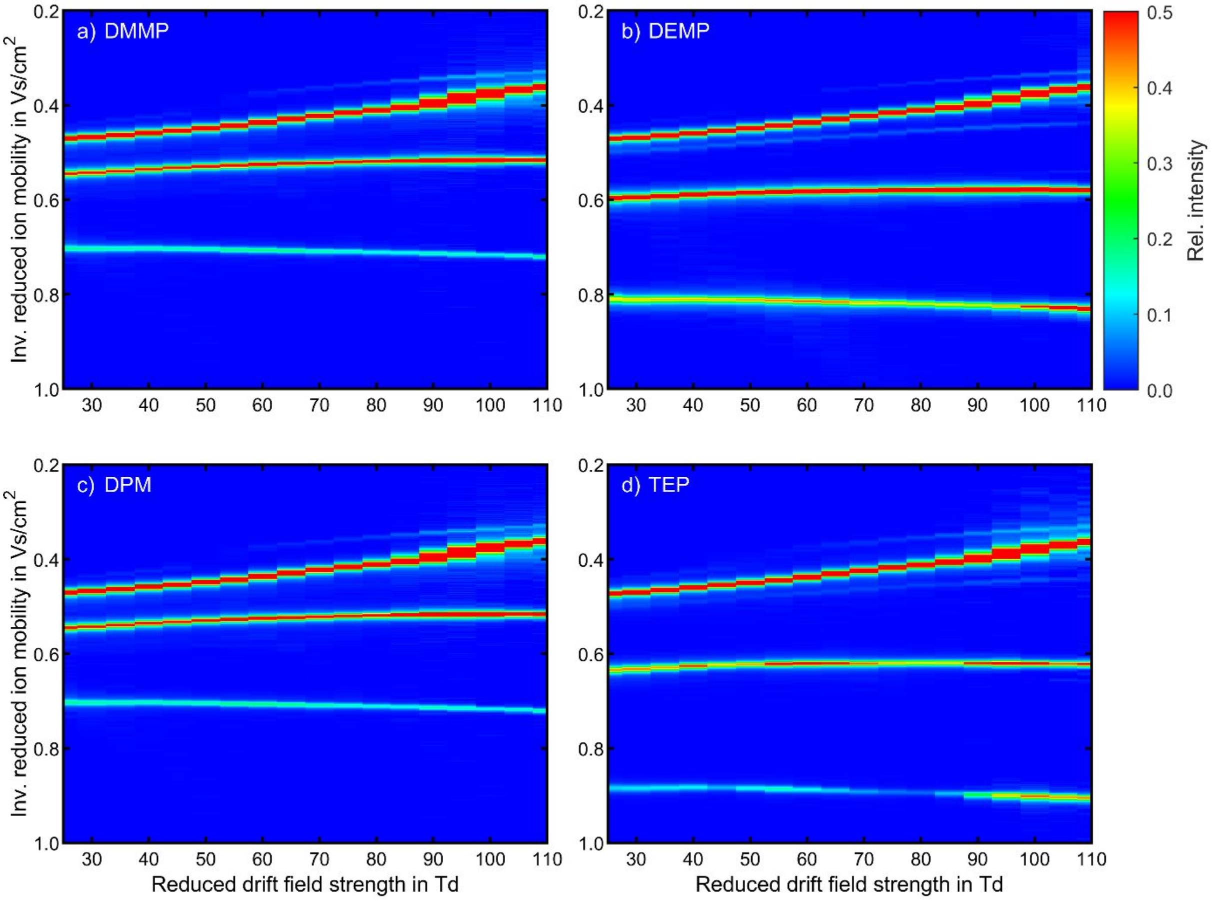

Figure S3. Dispersion plots of simulants a) dimethyl methylphosphonate (DMMP), b) diethyl methylphosphonate (DEMP), c) dipropylene glycol methyl ether (DPM) and d) triethyl phosphate (TEP) in positive polarity in dry air at E_{RR}/N = 25 Td. Each individual ion mobility spectrum is normalized to its maximum value of intensity. Each dispersion plot is scaled to 50 % of the maximum value for better visibility. All other operating parameters were set according to Table 1 in the main manuscript.

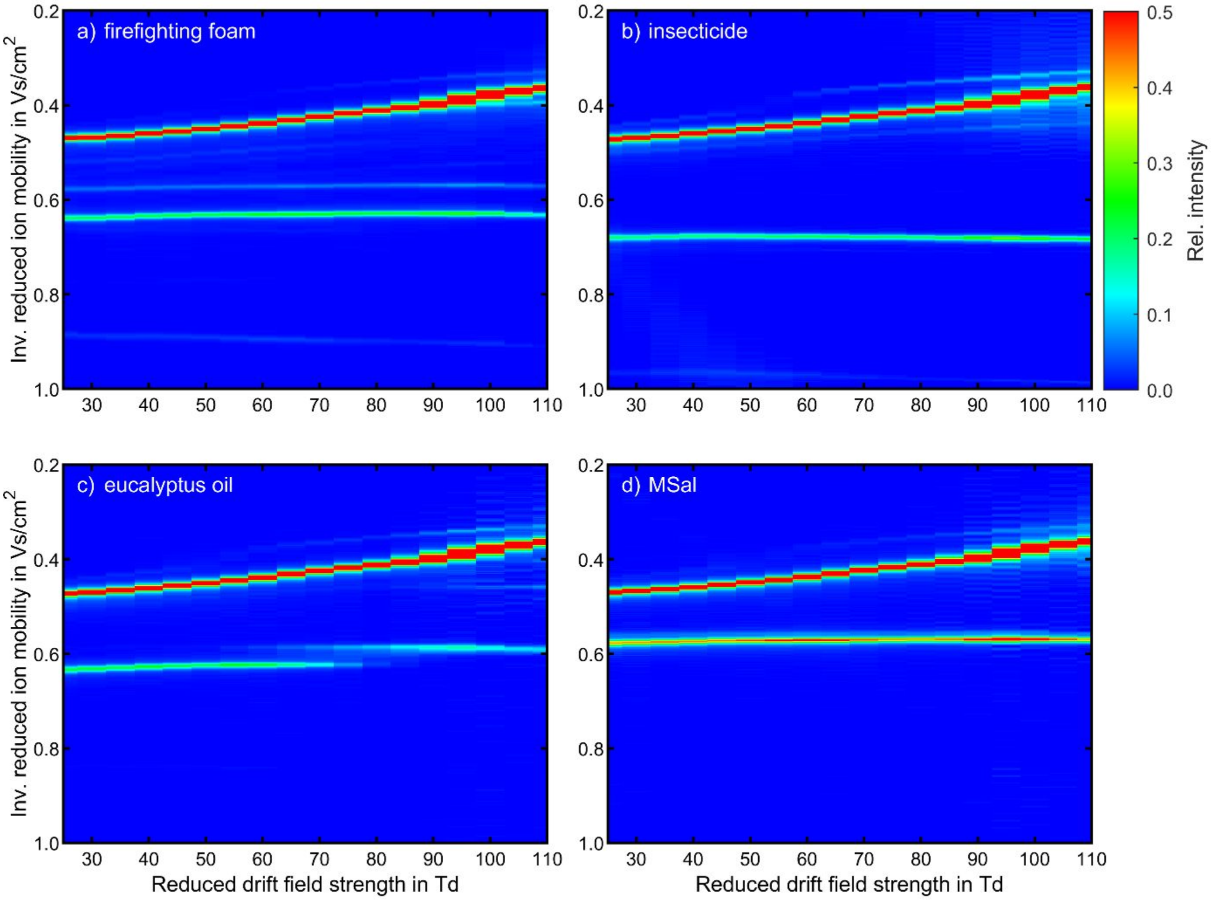

Figure S4. Dispersion plots of interferents a) firefighting foam, b) insecticide, c) eucalyptus oil and d) the simulant methyl salicylate (MSal) in positive polarity in dry air at E_{RR}/N = 25 Td. Each individual ion mobility spectrum is normalized to its maximum value of intensity. Each dispersion plot scaled to 50 % of the maximum value for better visibility. All other operating parameters were set according to Table 1 in the main manuscript.

Section S3. Sensitivity of HiKE-IMS

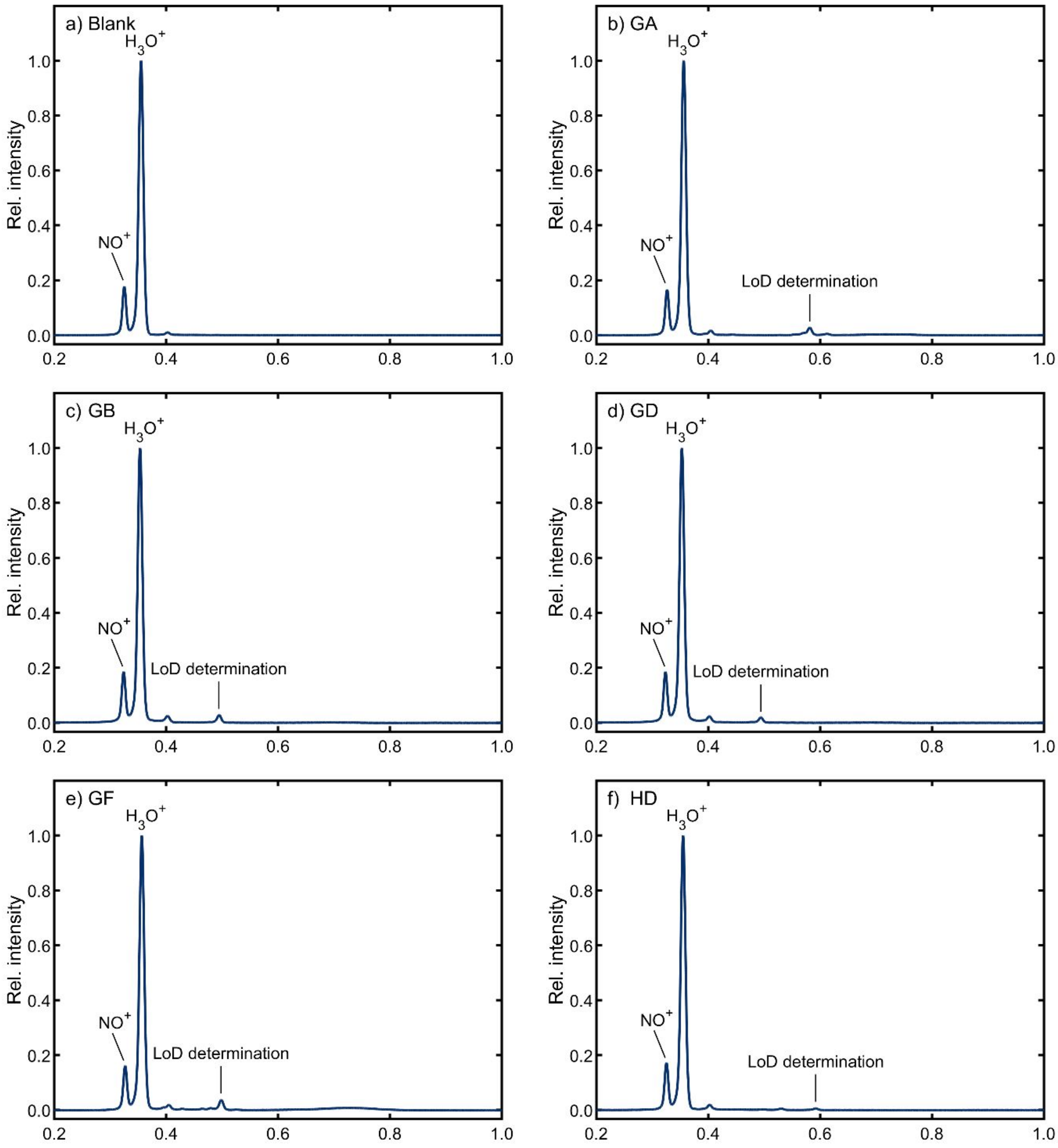

Figure S5. Ion mobility spectra of a) a blank measurement in comparison to chemical warfare agents b) 244 ppb_V GA, c) 216 ppb_V GB, d) 244 ppb_V GD, e) 217 ppb_V GF and f) 245 ppb_V HD in positive polarity in air with relative humidity of 50 % and at $E_{RR}/N = E_{DR}/N = 120$ Td. The peaks used for determination of the limit of detection are marked with 'LoD determination'. All other operating parameters were set according to Table 1 in the main manuscript.
